# Polymorphisms in Genes Involved in Osteoblast Differentiation and Function Are Associated with Anthropometric Phenotypes in Spanish Women

**DOI:** 10.3390/genes12122012

**Published:** 2021-12-17

**Authors:** Clara Pertusa, Sofía P. Ruzo, Layla Panach, Damián Mifsut, Juan J. Tarín, Antonio Cano, Miguel Ángel García-Pérez

**Affiliations:** 1 Research Unit, INCLIVA Health Research Institute, 46010 Valencia, Spain; Clara.Pertusa@uv.es (C.P.); sofiapirizruzo@gmail.com (S.P.R.); laylapanach@hotmail.com (L.P.); 2 Orthopedic Surgery and Traumatology, Clinic Hospital, INCLIVA Institute of Health Research, 46010 Valencia, Spain; mifsut.dam@gmail.com; 3 Department of Cellular Biology, Functional Biology and Physical Anthropology, University of Valencia, 46100 Burjassot, Spain; Juan.J.Tarin@uv.es; 4 Department of Pediatrics, Obstetrics and Gynecology, University of Valencia, 46010 Valencia, Spain; Antonio.Cano@uv.es; 5 Department of Genetics, University of Valencia, 46100 Burjassot, Spain

**Keywords:** women, osteoporosis, BMD, BMI, *CTNNB1* and *MEF2C* genes

## Abstract

Much of the genetic variance associated with osteoporosis is still unknown. Bone mineral density (BMD) is the main predictor of osteoporosis risk, although other anthropometric phenotypes have recently gained importance. The aim of this study was to analyze the association of SNPs in genes involved in osteoblast differentiation and function with BMD, body mass index (BMI), and waist (WC) and hip (HC) circumferences. Four genes that affect osteoblast differentiation and/or function were selected from among the differentially expressed genes in fragility hip fracture (*FOXC1*, *CTNNB1*, *MEF2C*, and *EBF2*), and an association study of four single-nucleotide polymorphisms (SNPs) was conducted in a cohort of 1001 women. Possible allelic imbalance was also studied for SNP rs87939 of the *CTNNB1* gene. We found significant associations of SNP rs87939 of the *CTNNB1* gene with LS-sBMD, and of SNP rs1366594 of the *MEF2C* gene with BMI, after adjustment for confounding variables. The SNP of the *MEF2C* gene also showed a significant trend to association with FN-sBMD (*p* = 0.009). A possible allelic imbalance was ruled out as no differences for each allele were detected in *CTNNB1* expression in primary osteoblasts obtained from homozygous women. In conclusion, we demonstrated that two SNPs in the *MEF2C* and *CTNNB1* genes, both implicated in osteoblast differentiation and/or function, are associated with BMI and LS-sBMD, respectively.

## 1. Introduction

Osteoporosis is a systemic metabolic skeletal disease associated with low bone mass and deterioration in bone microarchitecture, which significantly predisposes to fragility bone fracture. Bone mineral density (BMD) is the best available predictor of fragility fracture and is the parameter used by the WHO for osteoporosis diagnosis [1].

As a quantitative phenotype, BMD is determined by genetic, epigenetic, and environmental factors. Dozens of studies aimed at characterizing genetic factors have identified metabolic and transduction pathways involved in this phenotype, such as Wnt signaling, PTH signaling, ossification, osteoblast (OB)/osteoclast (OC) differentiation, OB/OC communication, TGF signaling, and myostatin signaling [2,3]. However, despite the numerous linkage and association studies carried out, to date, only a small fraction of total phenotypic variance has been explained [4]. As a result, this missing heritability has been a target of research for several years in low-frequency variants, structural variants, and gene–gene and gene–environment interactions, among others [5,6,7].

Our research has sought to identify new genes and polymorphisms associated with BMD, largely through functional models. One example is a model of accelerated bone loss using the ovariectomized mouse to reproduce the genetic, biochemical, and cellular processes that occur in menopausal women experiencing increased trabecular and, to a lesser extent, cortical bone loss [8,9]. In another functional approach designed to identify genes associated with BMD, our group analyzed differential gene expression of primary osteoblasts obtained from bone explants of women with fragility hip fracture compared to those of controls [10].

Several anthropometric parameters have recently been correlated to BMD and risk of fractures. As examples, studies have variously reported low BMD as associated with low body mass index (BMI) and high waist-to-hip ratio (WHR) [11], as well as height as positively associated with fracture [12]; moreover, a recent study using Mendelian randomization method found that increased height was associated with an increased risk of fracture and that BMI was causally linked with estimated BMD [13].

Following one of the functional approaches described, this study sought to analyze in a cohort of 1001 women the association of BMD together with other anthropometric parameters such as BMI and waist (WC) and hip (HC) circumferences with four SNPs in four genes involved in osteoblast differentiation and/or function (*FOXC1*, *CTNNB1*, *MEF2C*, and *EBF2*) whose expression differs between osteoblasts from patients with hip fracture and controls.

## 2. Materials and Methods

### 2.1. Study Subjects

In the present study, we analyzed a cohort of Caucasian women consecutively recruited in the menopause clinic of the Gynecology and Obstetrics department in two hospitals in Valencia, the Hospital Clínico and the Hospital Doctor Peset; this cohort has been routinely evaluated in women’s health studies [10,14,15].

The only inclusion criterion was attendance at the menopause clinic. Exclusion criteria were (i) bone disease other than primary osteoporosis; (ii) primary amenorrhea, hyperparathyroidism, hyperthyroidism, rheumatoid arthritis, Addison’s disease, hemochromatosis, hepatitis B or C, or serious neurological illness; (iii) chemotherapy before densitometric study; (iv) previous corticosteroid treatment; and (v) being under 35 years of age. Bone active treatment, such as hormone therapy (HT) or bisphosphonates, was considered as a covariate. A total of 1128 women agreed to participate in the study and signed informed consent. After applying exclusion criteria, 1001 women were finally included in the study.

The study was approved by the local institutional review boards in accordance with the principles of the Declaration of Helsinki, and written informed consent was obtained from participants in accordance with the of INCLIVA Biomedical Research Institute guidelines.

### 2.2. Biochemical Assays, and Anthropometric and Bone Mineral Density (BMD) Data

Baseline clinical data including blood test, bone active treatments, and relevant anthropometric data such as age, weight, height, menopausal status were obtained as previously described [9]. Levels of several bone metabolism markers such as carboxy-terminal telopeptides levels of collagen I (CTx); total alkaline phosphatase (ALP); hormones such as insulin, estradiol, and FSH; and other metabolites were quantified as previously described [10,15]. The HOMA-IR (homeostasis model assessment) insulin resistance index was calculated as (fasting serum insulin in µIU/mL) × (fasting serum glucose in mg/dL × 0.05551)/22.5.

To assess bone status, we performed densitometric studies at femoral neck (FN-BMD) and/or lumbar spine L2-L4 (LS-BMD) sites by dual-energy X-ray absorptiometry (DXA). As different densitometers were used during patient recruitment, we used a standardized BMD (sBMD) value in the present study [10].

### 2.3. Genes, Single-Nucleotide Polymorphisms (SNPs), and Genotyping

In the present study, we analyzed the genes differentially expressed in primary osteoblasts of women with bone fracture compared to controls [10]. We chose genes with a fold change ± 1.3 previously correlated in the literature with the bone cell function and/or differentiation, using this method to select the genes that are shown in Table 1.

*MEF2C* (myocyte-specific enhancer factor 2C) was selected for its involvement in osteocyte function [16]. *EBF2* (early B-cell factor 2) is related to the osteoblast-dependent differentiation of osteoclasts [17]. *FOXC1* (forkhead box C1) is implicated in initiation of early osteoblast differentiation [18], while *CTNNB1* (catenin beta 1) is involved in the pathway that bears its name (Wnt/β-catenin), determinant in osteoblast formation [19]. Of these genes, only *MEF2C* and *CTNNB1* have previously been related to BMD in humans [20,21].

Single nucleotide polymorphisms (SNPs) were chosen on the basis of minor allele frequency (MAF) > 5% in the Caucasian population and positive correlation with BMD in previous studies (*CTNNB1* and *MEF2C*). In the case of *FOXC1* and *EBF2* genes, SNPs that captured a reasonable number of SNPs in the genomic region were selected using the LDlink web tool (https://ldlink.nci.nih.gov/, accessed on 2 November 2020).

DNA extraction and purification, SNP genotyping by allelic discrimination using TaqMan SNP Genotyping Assays (Thermo Fisher, Waltham, MA, USA), and validity controls were performed as previously described [22].

### 2.4. Functionality of rs87939 in CTNNB1 by Gene-Reporter Assay

A region of 336 bp was amplified from gDNA of genotyped women from our cohort between positions chr3: 41,096,240-41,096,575 (GRCh38.p12), which contains SNP rs87939. The primers used were designed with primer-3 (http://bioinfo.ut.ee/primer3-0.4.0/primer3/, accessed on 12 July 2021) and target sequences (underlined) for restriction endonucleases, and a poly(A) tail were added to aid digestion. Primer sequences were 5′-aaaaaagagctcCACCTCCTCAGTGACTGCAA-3′ for the forward primer, with a target sequence for *Sac*I (5′-GAGCTC-3′), and 5′-aaaaaactcgagAAAGCCTGCAGGATCAGAAA-3′ for the reverse primer, with a target sequence for *Xho*I (5′-CTCGAG-3′).

Samples of gDNA homozygotic for each of the two SNP alleles were selected for amplification then digested with *Sac*I and *Xho*I (New England Biolabs, Ipswich, MA, USA). The DNA inserts were next ligated into the PGL3-Promoter vector (Promega, Madison, WI, USA) using T4 ligase (Roche, Basel, Switzerland). Recombinant vectors were introduced in Escherichia coli via transformation by heat shock, and after overnight culture were purified with QIAGEN Plasmid Plus Maxi kit (Qiagen GmbH, Hilden, Germany).

MC3T3-E1 cells, a murine pre-osteoblastic cell line, were plated in 24-well plates and cultured until 80% confluence in DMEM medium (Invitrogen, Waltham, MA, USA) with 10% FCS and antibiotics (complete medium). Cells in each well were co-transfected with 950 ng of reporter constructs or control plasmids and 47.5 ng of normalizing pRLSV40 vector (Promega) using Lipofectamine LTX with Plus Reagent (Thermo Fisher) following the manufacturer’s instructions. Cells were incubated in complete medium for 48 h, then collected, and luciferase activity was assayed using the Dual-Luciferase Reporter Assay System kit (Promega) with an X4 Victor Multilabel Plate Reader with luminescence detector (PerkinElmer, Waltham, MA, USA). Luciferase activity of PGL3 vectors was normalized against luciferase activity of the pRLSV40 vector, as indicated by the manufacturer.

### 2.5. Osteoblastic CTNNB1 Expression

Allele dependent *CTNNB1* expression for rs87939 was determined in osteoblasts from 7 women with GG genotype and 7 women with AA genotype. Primary OBs were obtained from Caucasian women undergoing hip replacement surgery at Hospital Clínico Universitario de Valencia, due to either subcapital hip osteoporotic fracture or severe hip osteoarthritis. Osteoblastic cultures were retrieved from the femoral head, and their lineage was confirmed using flow cytometry as described in [10].

Total RNA was extracted from OBs using TRIzol reagent (Invitrogen) according to the manufacturer’s instructions. RNA concentration was determined using NanoDrop One (Thermo Fisher), and 200 ng of each sample was used for reverse transcription (RT). The reaction was performed using Maxima H Minus First Strand cDNA Synthesis Kit (Thermo Fisher) following the manufacturer’s instructions. Quantitative real-time amplification of *CTNNB1*, and *ACTB* and *GAPDH* as housekeeping was performed using TaqMan Fast Advanced Master Mix (Thermo Fisher) and the specific primers and probe for each gene present in TaqMan Gene Expression Assay (Thermo Fisher). We used the mean Ct value of two housekeeping genes for the normalization in order to minimize possible variations in gene expression. The reaction was carried out using QuantStudio 5 Real-Time PCR System (Thermo Fisher), and amplification curves were analyzed with QuantStudio Design and Analysis Software (Thermo Fisher).

### 2.6. Statistical Analysis

In this study, we used the SNPStats software (https://www.snpstats.net/start.htm, accessed on 29 September 2021) to contrast the genotype frequencies of our population with those expected according to the Hardy–Weinberg equilibrium (HWE) and to estimate model inheritance (co-dominant, dominant, overdominant, or recessive) [23].

To minimize the effect of outliers detected by Tukey’s test, we winsorized quantitative biochemical, bone, and anthropometric variables (i.e., the data point was replaced with the next highest or lowest non-outlier value). To adjust for potential biases due to missing quantitative values, although we assumed missing at random, we considered five imputations and pooled estimates of these imputations used for statistical analyzes.

Fixed-effects analysis of variance (ANOVA) designs were used to compare means between genotypes. Analysis of covariance (ANCOVA) was used to explore differences in dependent variables among genotypes after adjustment for confounding variables. Age and BMI were considered as covariates. Postmenopausal status (yes/no) and antiresorptive therapy use (yes/no) were introduced as dummy variables, which were codified as 0 or 1.

In the present study, a sample size of 950 subjects was estimated to reach a statistical power of 90% using the QUANTO software (http://hydra.usc.edu/GxE/, accessed on 4 October 2021). The data entered in the statistical tool were (i) gene effect of R^2^ = 0.011 obtained by regression analysis between LS-BMD and rs87939, (ii) G allele frequency of 0.47 (Table 1), (iii) recessive model of inheritance, and (iv) mean ± SD for LS-BMD of 0.989 ± 0.150 (Table 2).

Statistical significance was defined as *p* < 0.05, except for multiple comparisons, in which case the cut-off value for the Bonferroni correction was estimated as *p* < 0.0025 (0.05/20; five phenotypes—LS-sBMD, FN-sBMD, HC, WC, and BMI—and four SNPs). All analyses were two-tailed. Data were analyzed using IBM SPSS statistics for Windows (v.26.0; IBM Corp, Armonk, NY, USA).

## 3. Results

Table 1 shows the SNPs analyzed in this study. The allele frequencies agreed with those published for the IBS population (Iberian populations in Spain; Ensembl genome browser), and the genotype frequencies met HWE.

Table 2 shows the anthropometric and bone characteristics of study subjects, a group defined by very mild osteopenia consistent with mean age.

A previous ANOVA study of the four SNPs with the phenotypes FN-sBMD, LS-sBMD, HC, WC, and BMI (not shown) all showed either a clear trend (*p* < 0.1) or nominally significant association (*p* < 0.05) with BMD, while the SNP of the *MEF2C* gene was significantly associated with BMI, WC, and HC.

Table 3 shows the association of SNPs with BMD according to the inheritance model, with or without adjustment for covariates. Although several associations are described with a nominal *p*-value < 0.05, only one (the SNP of the *CTNNB1* gene with LS-sBMD) remained significant after applying the Bonferroni threshold (*p* = 0.0025), although the association of the SNP in *MEF2C* gene to FN-sBMD came very close (*p* = 0.009) to statistical significance.

Regarding anthropometric parameters (Table 4), the SNP of the *MEF2C* gene showed a significant association (*p* = 0.001) with BMI, while the significant associations with WC and HC were lost when adjusting for age and BMI covariates.

Table 5 shows biochemical and bone characteristics of subjects by *CTNNB1* and *MEF2C* genotypes. The different genotypes did not differ significantly in terms of bone metabolism markers, lipids, and insulin resistance, which precludes attributing the changes in BMD or BMI described in this work to differences in insulin resistance or in lipid or bone metabolism. However, we did detect different levels of estradiol and FSH between the genotypes of the *MEF2C* and *CTNNB1* genes, respectively.

**Figure 1:**
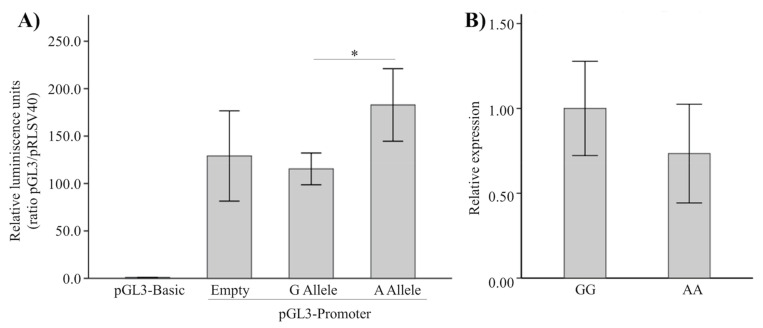
(**A**) Relative luciferase activity in MC3T3 cells. Values were normalized against the luciferase activity of Renilla vector pRLSV40. The G and A allele vectors were made in pGL3-Promoter by cloning a 336 bp region containing the SNP rs87939. Alleles G and A showed a statistically significant difference in luciferase activity levels (*p*-value = 0.007). (**B**) *CTNNB1* expression (mean ± SE) in primary OBs according to genotype of SNP rs87939. Gene expression was calculated with the 2^−ΔΔCt^ formula, with ΔCt representing the difference between the *CTNNB1* Ct and the mean of the housekeeping genes Cts (*ACTB* and *GAPDH*). Values were normalized for gene expression of the GG genotype. * *p*-value = 0.007.

We next examined the possible functionality of rs87939 on the *CTNNB1* gene, and given that the distance between these elements is 103 Kb, we cloned both alleles in the PGL3-Promoter vector to check whether this polymorphism could be found in a transcriptional regulatory sequence (Figure 1A). As can be seen, the A allele stimulated luciferase production to a greater extent than the G allele, although neither allele differed in expression from the expression of the empty PGL3-Promoter vector.

To determine whether these in vitro results translated into the in vivo expression rate, we decided to study a possible allelic imbalance. To do this, we analyzed *CTNNB1* expression in total RNA from primary osteoblasts obtained from seven homozygous women for each allele (Figure 1B). As can be observed, gene expression did not differ between alleles, and therefore we conclude that the SNP rs87939 is not an eQTL, at least with regards to the *CTNNB1* gene.

## 4. Discussion

In this study, we describe the significant association, adjusted for confounding variables, of SNP rs87939 of the *CTNNB1* gene to LS-sBMD and of SNP rs1366594 of the *MEF2C* gene to BMI, although the latter polymorphism also showed an important trend of association with FN-sBMD (*p* = 0.009). The small differences detected in the levels of estradiol and FSH between the genotypes of the *MEF2C* and *CTNNB1* genes, respectively (Table 5), did not modify the ANCOVA analysis (Table 3 and Table 4), indicating a very limited or null contribution to the phenotypic variation (not shown). To our best knowledge, this is the first reported association of genetic variation in *MEF2C* with BMI.

The main aim of this study was to identify possible associations between anthropometric phenotypes such as BMD, BMI, HC, and WC with SNPs in genes related to osteoblast differentiation and function. The genes were selected from the list of genes differentially expressed in osteoblasts from a previously published study of women with fragility hip fracture [10]. Although in this study we detected important trends, or even nominal significance (*p* < 0.05) of the SNPs in the *EBF2* and *FOXC1* genes with respect to BMD, none were significant after adjusting for covariates (Table 3).

The catenin beta 1 protein encoded by the *CTNNB1* gene is a determining factor in osteoblastic differentiation [19]. According to Ensembl (https://www.ensembl.org, accessed on 20 October 2021) and the Musculoskeletal Knowledge Portal (http://mskkp.org/, accessed on 20 October 2021), the rs87939 polymorphism of the *CTNNB1* gene has previously been associated with FN- and LS-BMD, height, osteoporosis, and bone fracture. In our study, this variant was not associated with FN-sBMD, since despite obtaining nominal significance (*p* < 0.05), it failed to pass the Bonferroni threshold established in this study at *p* = 0.0025. 

MEF2C is a transcription factor of the MEF2 family encoded by the *MEF2C* gene that is necessary for correct embryonic development and regulation of muscle development [24]. SNP rs1366594 in this gene has previously been linked with height, BMD, and male-pattern baldness, but not with BMI. In this study, this variant was also associated with unadjusted WC and HC, but when we adjusted for BMI and age, the significance was lost, indicating that the primary variable is BMI and that the statistical association of rs1366594 to WC and HC is secondary to its association with BMI [25].

Neither of the two SNPs in the *MEF2C* or *CTNNB1* genes appear as an expression quantitative trait locus (eQTL) in the GTExPortal (www.gtexportal.org/, accessed on 20 October 2021). Despite this, in the present work, we searched for a possible allelic imbalance for the SNP rs87939 of the *CTNNB1* gene (Figure 1). Since no allele-dependent differential expression of the *CTNNB1* gene was found, our data support the GTExPortal data in that this genetic variant is not an eQTL, at least with respect to the *CTNNB1* gene.

Despite the considerable statistical power and strengths, our study must be interpreted in light of several limitations. This is a study in women, and therefore its translation to men has yet to be demonstrated; likewise, we analyzed only one SNP of each gene, and therefore we cannot capture all the genetic variance of each gene. Finally, another possible limitation of our study is that osteoarthritis-related genes were found in the list of genes differentially expressed in fracture, since the controls were women who needed hip replacement due to severe osteoarthritis [10]. Nonetheless, the osteoblasts were obtained from trabecular bone, far from the joint surface, and thus the transcriptomic signatures obtained will pertain mostly to osteoporosis rather than osteoarthritis.

In conclusion, in the present work, we demonstrated that two SNPs in the *MEF2C* and *CTNNB1* genes, both implicated in differentiation and/or function of osteoblasts, are associated with BMI and LS-sBMD, respectively. According to our own functional results and public gene expression data, the SNPs studied did not appear to be eQTLs, and therefore must be in linkage disequilibrium with the causal variants.

## Figures and Tables

**Table 1:** Genes and SNPs studied.

Chromosome	Gene	Fold Change ^1^	SNP	Position (GRCh38.p12)	Location	Major Allele	Minor Allele	MAF	*p*-HWE ^2^
3	*CTNNB1*	1.3	rs87939	41096353	Intergenic	A	G	0.47	0.70
8	*EBF2*	−1.79	rs9314318	26046872	2 KB Upstream Variant	G	A	0.25	0.21
6	*FOXC1*	1.64	rs2745599	1613451	3′UTR Variant	A	G	0.45	0.14
5	*MEF2C*	1.55	rs1366594	89080244	Intronic	A	C	0.49	0.95

^1^ Data taken from Panach et al. [10]. ^2^ *p*-values were obtained from chi-squared test.

**Table 2:** Anthropometric and bone characteristics of the cohort studied. Values are mean ± SD or percentage. N = 1001 women ^1^.

	Values
Age (y)	55.7 ± 8.7
Weight (kg)	66.5 ± 10.3
Height (cm)	157.5 ± 6.0
BMI (kg/m^2^)	26.8 ± 4.1
Waist circumference (cm)	85.9 ± 10.4
Hip circumference (cm)	102.4 ± 8.2
Postmenopausal women (%)	97.3
Antiresorptive therapy user (%)	25.3
FN-sBMD (g/cm^2^)	0.795 ± 0.117
FN T-score	−1.006 ± 0.994
FN Z-score	−0.081 ± 0.926
LS-sBMD (g/cm^2^)	0.989 ± 0.150
LS T-score	−1.207 ± 1.331
LS Z-score	−1.121 ± 1.227

^1^ For percentage of postmenopausal women, N = 950, and for percentage of antiresorptive therapy users, N = 946. BMI, body mass index; sBMD, standardized bone mineral density; FN, femoral neck; LS, lumbar spine.

**Table 3:** Association of SNPs, according to the inheritance model, with FN- and LS-sBMD, with and without adjustment for age, BMI, postmenopausal status, and treatment use. Unadjusted values are means ± SD. Adjusted values are estimated means ± SE.

Gene SNP (rs)	Genotype (N)	FN-sBMD		Genotype (N)	LS-sBMD	
		Unadjusted	Adjusted		Unadjusted (N)	Adjusted (N)
***CTNNB1*** rs87939	A/A (284)	0.807 ± 0.123	0.807 ± 0.007	A/A (284)	1.014 ± 0.162	1.049 ± 0.017
	A/G–G/G (716)	0.791 ± 0.114	0.789 ± 0.004	A/G–G/G (716)	0.979 ± 0.144	1.015 ± 0.015
	*p*-value	0.047	0.020	*p*-value	0.0009	0.0017
***EBF2*** rs9314318	A/A (55)	0.823 ± 0.108	0.813 ± 0.016	A/G (391)	0.996 ± 0.150	1.034 ± 0.016
	A/G–G/G (939)	0.794 ± 0.117	0.793 ± 0.004	A/A–G/G (603)	0.984 ± 0.150	1.017 ± 0.015
	*p*-value	0.073	0.210	*p*-value	0.211	0.079
***FOXC1*** rs2745599	G/G (218)	0.808 ± 0.113	0.805 ± 0.008	G/G (218)	1.009 ± 0.149	1.025 ± 0.019
	A/G–A/A (779)	0.792 ± 0.118	0.791 ± 0.004	A/G–A/A (779)	0.983 ± 0.149	1.013 ± 0.016
	*p*-value	0.066	0.114	*p*-value	0.023	0.407
***MEF2C*** rs1366594	A/A (260)	0.808 ± 0.127	0.876 ± 0.028	A/A (260)	0.998 ± 0.153	1.103 ± 0.037
	A/C–C/C (729)	0.791 ± 0.113	0.798 ± 0.012	A/C–C/C (729)	0.986 ± 0.149	1.009 ± 0.016
	*p*-value	0.042	0.009	*p*-value	0.252	0.020

**Table 4:** Association of *MEF2C* SNP, according to the inheritance model, with BMI, and waist (WC) and hip (HC) circumference. WC and HC were adjusted for BMI and age, whereas BMI was adjusted for age. Unadjusted values are means ± SD. Adjusted values are estimated means ± SE.

Gene SNP (rs)	Genotype (N)	Waist Circumference		Hip Circumference		BMI	
		Unadjusted	Adjusted	Unadjusted	Adjusted	Unadjusted	Adjusted
***MEF2C*** rs1366594	A/A (260)	84.1 ± 9.7	85.6 ± 0.4	101.0 ± 8.0	102.1 ± 0.3	26.1 ± 3.9	26.1 ± 0.2
	A/C–C/C (729)	86.5 ± 10.6	85.9 ± 0.2	102.9 ± 8.3	102.5 ± 0.2	27.1 ± 4.1	27.1 ± 0.1
	*p*-value	0.0018	0.386	0.0014	0.386	0.0011	0.0010

**Table 5:** Biochemical and bone characteristics of subjects according to rs87939 (*CTNNB1*) and rs1366594 (*MEF2C*) genotypes.

	rs87939 (*CTNNB1*)		*p*	rs1366594 (*MEF2C*)		*p*
	AA (N = 284)	AG/GG (N = 716)		AA (N = 260)	AC/CC (N = 729)	
Age (y)	55.7 ± 9.0	55.6 ± 8.5	0.915	55.7 ± 9.1	55.7 ± 8.5	0.998
BMI (kg/m^2^)	27.0 ± 4.2	26.8 ± 4.1	0.495	26.1 ± 3.9	27.1 ± 4.1	0.0011
FN-BMD (g/cm^2^)	0.807 ± 0.124	0.791 ± 0.114	0.047	0.808 ± 0.127	0.791 ± 0.113	0.042
FN Z-score	0.040 ± 0.946	−0.130 ± 0.914	0.009	0.038 ± 0.953	−0.121 ± 0.916	0.018
LS-BMD (g/cm^2^)	1.014 ± 0.162	0.979 ± 0.144	0.0009	0.998 ± 0.153	0.986 ± 0.149	0.252
LS Z-score	0.094 ± 1.279	−0.209 ± 1.193	0.0004	−0.023 ± 1.250	−0.153 ± 1.219	0.142
Estradiol (pg/mL)	16.5 ± 25.2	14.7 ± 8.4	0.089	17.1 ± 26.2	14.5 ± 8.4	0.021
FSH (U/mL)	69.7 ± 29.1	73.8 ± 27.1	0.039	71.3 ± 30.7	73.0 ± 26.8	0.410
25(OH)D3 (ng/mL)	26.1 ± 9.4	25.6 ± 9.3	0.479	25.7 ± 8.5	25.8 ± 9.6	0.877
CTx (ng/mL)	0.436 ± 0.205	0.439 ± 0.190	0.793	0.448 ± 0.203	0.434 ± 0.192	0.346
Total ALP (U/L)	168.9 ± 52.0	165.8 ± 46.3	0.364	167.7 ± 48.5	166.6 ± 47.7	0.755
Calcium (mg/dL)	9.6 ± 0.4	9.6 ± 0.4	0.326	9.6 ± 0.4	9.6 ± 0.4	0.475
Phosphate (mg/dL)	3.6 ± 0.5	3.6 ± 0.5	0.722	3.6 ± 0.5	3.6 ± 0.5	0.425
Triglycerides (mg/dL)	102.9 ± 39.6	100.7 ± 39.4	0.430	99.7 ± 37.3	101.7 ± 40.4	0.496
Total cholesterol (mg/dL)	214.8 ± 34.0	215.8 ± 35.1	0.691	216.4 ± 33.7	215.1 ± 35.3	0.613
HDL-cholesterol (mg/dL)	64.3 ± 14.0	64.2 ± 14.1	0.946	64.5 ± 14.0	64.2 ± 14.2	0.747
LDL-cholesterol (mg/dL)	129.9 ± 29.1	131.8 ± 29.1	0.330	132.2 ± 27.3	130.8 ± 29.8	0.502
Glucose (mg/dL)	100.9 ± 11.7	99.9 ± 11.5	0.209	99.5 ± 10.7	100.3 ± 11.9	0.304
Insulin (μU/mL)	8.4 ± 4.7	8.5 ± 5.2	0.762	8.2 ± 4.2	8.5 ± 5.4	0.359
HOMA-_IR_ index	2.1 ± 1.4	2.2 ± 1.6	0.878	2.1 ± 1.2	2.2 ± 1.6	0.279

## Data Availability

The data are not publicly available due to privacy or ethical restrictions. The data that support the findings of this study are available from the corresponding author upon reasonable request and approval by the ethics committee of our hospital.

## References

[1] Kanis J.A., Cooper C., Rizzoli R., Reginster J.-Y.Y., McCloskey E.V., Johansson H., Cooper C., Rizzoli R., Reginster J.-Y.Y., McCloskey E.V. (2019). Executive summary of the european guidance for the diagnosis and management of osteoporosis in postmenopausal women. Calcif. Tissue Int..

[2] Saad F.A. (2020). Novel insights into the complex architecture of osteoporosis molecular genetics. Ann. N. Y. Acad. Sci..

[3] Hsu Y.H., Kiel D.P. (2012). Genome-wide association studies of skeletal phenotypes: What we have learned and where we are headed. J. Clin. Endocrinol. Metab..

[4] Morris J.A., Kemp J.P., Youlten S.E., Laurent L., Logan J.G., Chai R.C., Vulpescu N.A., Forgetta V., Kleinman A., Mohanty S.T. (2019). An atlas of genetic influences on osteoporosis in humans and mice. Nat. Genet..

[5] Zheng H.F., Forgetta V., Hsu Y.-H., Estrada K., Rosello-Diez A., Leo P.J., Dahia C.L., Park-Min K.H., Tobias J.H., Kooperberg C. (2015). Whole-genome sequencing identifies EN1 as a determinant of bone density and fracture. Nature.

[6] Park S., Daily J.W., Song M.Y., Kwon H.-K. (2020). Gene-gene and gene-lifestyle interactions of AKAP11, KCNMA1, PUM1, SPTBN1, and EPDR1 on osteoporosis risk in middle-aged adults. Nutrition.

[7] Costantini A., Skarp S., Kämpe A., Mäkitie R.E., Pettersson M., Männikkö M., Jiao H., Taylan F., Lindstrand A., Mäkitie O. (2018). Rare copy number variants in array-based comparative genomic hybridization in early-onset skeletal fragility. Front. Endocrinol..

[8] Pineda B., Serna E., Laguna-Fernández A., Noguera I., Panach L., Hermenegildo C., Tarín J.J.J., Cano A., García-Pérez M.Á., Garcia-Perez M. (2014). Gene expression profile induced by ovariectomy in bone marrow of mice: A functional approach to identify new candidate genes associated to osteoporosis risk in women. Bone.

[9] Panach L., Serna E., Tarín J.J., Cano A., García-Pérez M.Á. (2017). A translational approach from an animal model identifies CD80 as a candidate gene for the study of bone phenotypes in postmenopausal women. Osteoporos. Int..

[10] Panach L., Pertusa C., Martínez-Rojas B., Acebron A., Mifsut D., Tarín J.J., Cano A., Garcia-Perez M.A. (2020). Comparative transcriptome analysus identifies CARM1 and DNMT3A as genes associated with osteoporosis. Sci. Rep..

[11] Hasani-Ranjbar S., Jafari-Adli S., Payab M., Qorbani M., Ahanjideh F., Keshtkar A., Larijani B. (2019). Association of osteoporosis with anthropometric measures in a representative sample of iranian adults: The iranian multicenter osteoporosis study. Int. J. Prev. Med..

[12] Armstrong M.E., Kirichek O., Cairns B.J., Green J., Reeves G.K. (2016). Relationship of height to site-specific fracture risk in postmenopausal women. J. Bone Miner. Res..

[13] Ma B., Li C., Pan J., Zhang S., Dong H., Wu Y., Lv J. (2021). Causal associations of anthropometric measurements with fracture risk and bone mineral density: A mendelian randomization study. J. Bone Miner. Res..

[14] Pineda B., Pertusa C., Panach L., Tarín J.J., Cano A., García-Pérez M.Á. (2020). Polymorphisms in genes involved in T-cell co-stimulation are associated with blood pressure in women. Gene.

[15] Zolfaroli I., Ortiz E., García-Pérez M.Á., Hidalgo-Mora J.J., Tarín J.J., Cano A. (2021). Positive association of high-density lipoprotein cholesterol with lumbar and femoral neck bone mineral density in postmenopausal women. Maturitas.

[16] Wein M.N., Spatz J., Nishimori S., Doench J., Root D., Babij P., Nagano K., Baron R., Brooks D., Bouxsein M. (2015). HDAC5 controls MEF2C-driven sclerostin expression in osteocytes. J. Bone Miner. Res..

[17] Kieslinger M., Folberth S., Dobreva G., Dorn T., Croci L., Erben R., Consalez G.G., Grosschedl R. (2005). EBF2 regulates osteoblast-dependent differentiation of osteoclasts. Dev. Cell.

[18] Mirzayans F., Lavy R., Penner-Chea J., Berry F.B. (2012). Initiation of early osteoblast differentiation events through the direct transcriptional regulation of Msx2 by FOXC1. PLoS ONE.

[19] Day T.F., Guo X., Garrett-Beal L., Yang Y. (2005). Wnt/Beta-catenin signaling in mesenchymal progenitors controls osteoblast and chondrocyte differentiation during vertebrate skeletogenesis. Dev. Cell.

[20] Rivadeneira F., Styrkársdottir U., Estrada K., Halldórsson B.V., Hsu Y.-H., Richards J.B., Zillikens M.C., Kavvoura F.K., Amin N., Aulchenko Y.S. (2009). Twenty bone-mineral-density loci identified by large-scale meta-analysis of genome-wide association studies. Nat. Genet..

[21] Zheng H.-F., Duncan E.L., Yerges-Armstrong L.M., Eriksson J., Bergström U., Leo P.J., Leslie W.D., Goltzman D., Blangero J., Hanley D.A. (2013). Meta-analysis of genome-wide studies identifies MEF2C SNPs associated with bone mineral density at forearm. J. Med. Genet..

[22] Panach L., Mifsut D., Tarín J.J., Cano A., García-Pérez M.Á. (2014). Replication study of three functional polymorphisms associated with bone mineral density in a cohort of spanish women. J. Bone Miner. Metab..

[23] Sole X., Guino E., Valls J., Iniesta R., Moreno V. (2006). SNPStats: A web tool for the analysis of association studies. Bioinformatics.

[24] Lin Q., Schwarz J., Bucana C., Olson E.N. (1997). Control of mouse cardiac morphogenesis and myogenesis by transcription factor MEF2C. Science.

[25] Heard-Costa N.L., Carola Zillikens M., Monda K.L., Johansson Å., Harris T.B., Fu M., Haritunians T., Feitosa M.F., Aspelund T., Eiriksdottir G. (2009). NRXN3 is a novel locus for waist circumference: A genome-wide association study from the CHARGE consortium. PLoS Genet..

